# Relationships between actual and desired workplace characteristics and job satisfaction for community health workers in China: a cross-sectional study

**DOI:** 10.1186/s12875-014-0180-y

**Published:** 2014-11-18

**Authors:** Li Li, Zhong Zhang, Zhinan Sun, Hao Zhou, Xinyan Liu, Heng Li, Lihua Fan, Peter C Coyte

**Affiliations:** Department of Health Management, School of Public Health, Harbin Medical University, Harbin, People’s Republic of China; English Department, School of Humanities and Social Science, Harbin, People’s Republic of China; Emergency Office, Harbin Center for Disease Control and Prevention, Harbin, People’s Republic of China; Institute of Health Policy, Management & Evaluation, University of Toronto, Toronto, Canada

**Keywords:** Actual workplace characteristics, Desired workplace characteristics, Job satisfaction, China

## Abstract

**Background:**

Community health workers are the main providers of community health services in China and have been important in the process of health system reform that has been in place since 2009. Therefore, it is critical that healthcare managers and policy decision makers motivate current staff and improve their job satisfaction. This study examined workplace characteristics and their contribution to job satisfaction in community health workers in Heilongjiang Province, China.

**Methods:**

A cross-sectional survey of 448 community health workers, from three cities in Heilongjiang province, was conducted between October 1, 2012 and December 31, 2012. Multistage sampling procedures were used to measure socioeconomic and demographic status, job satisfaction, and both actual and desired workplace characteristics. Factor analysis was conducted to determine the main factors contributing to workplace characteristics, and multiple linear regression analysis was performed to assess the key determinants of job satisfaction.

**Results:**

Eight groups of factors were identified as the most important workplace characteristics. These comprised system and policy; fringe benefits; work itself; work relationships; professional development; recognition; work environment; and remuneration. In all cases, all desired workplace characteristics were higher than the associated actual workplace characteristics. The main determinants of job satisfaction were occupation, years worked in health service institution, and five subscales representing the gap between desired and actual workplace characteristics, which were system and policy; fringe benefits; working relationship; professional development; and remuneration.

**Conclusions:**

These findings suggested that managers wishing to enhance job satisfaction should assess workplace characteristics comprehensively and design mechanisms that reduce the gap between actual and desired workplace characteristics.

## Background

China introduced the concept of general practice in the 1980s and began to build community health services in urban areas in the 1990s. In 2009, a new set of health system reforms, officially introduced by China’s central government, called for the development of community health services. The state established basic public health service items, which focused on providing basic public health services for urban and rural residents. These services were provided by general practitioners, nurses, and public health physicians from community health centers (CHCs) and community health stations.

Heilongjiang Province is located in Northeast China and has a population of approximately 38.1 million. There were 410 urban CHCs and 366 community health stations as of December 31, 2012 [1]. The integrated management of CHCs and their affiliated community health stations was adopted by the Heilongjiang Provincial Health Bureau, providing unified management to CHC and community health station employees.

There are 5,416 general practitioners working in community health institutions in the province. When this number is compared to the reference population, based on human resource planning ratios, there is a shortfall of approximately 30% in the number of general practitioners (5,416 vs. 7,620) [2]. Since the introduction of CHCs, these institutions have encountered difficulties due to limited resources, an insufficiency of staff members, and inadequate staff training [3]. Recent reforms have expanded the scope of public health services and increased workload without increasing the number of staff members [4,5]. Moreover, it has been demonstrated that work motivation can influence job satisfaction, which influences job performance [6-9]. Consequently, it is critical that healthcare managers and policy decision makers motivate current staff and improve their job satisfaction.

Increasing workers’ job satisfaction is a topic that has been studied extensively by researchers and managers [10]. Job satisfaction refers to the relationship between what workers desire from their jobs and their perceptions regarding what their jobs offer [11]. Some researchers have also proposed that job satisfaction is a function of the discrepancy between needs and outcomes [12-14]. In addition, job satisfaction can affect job performance, retention, and turnover [15-17]. Therefore, it is important that managers understand their employees’ needs, how the discrepancy between those needs and perceived incentives relates to job satisfaction, and how to motivate employees and increase their job satisfaction.

There are various workplace incentive policies in existence, such as those of financial and in-kind rewards, professional development, training opportunities, and positive working environments [18-21]. The value of an incentive depends on its actual content and the extent to which it matches what is desired by individuals [22,23]. There is extensive literature concerning factors affecting job satisfaction and motivation. Herzberg’s research results created the dual-factor theory of motivation [24]. Content theories were developed to link employee motivation and desired satisfaction [25]. Existence, relatedness, growth (ERG) theory, which reduced Maslow’s five levels of need to three categories (Existence, Relatedness, and Growth), was proposed by Clayton P. Alderfer [26]. Alderfer maintained that the three ERG areas are not hierarchical levels, and an employee’s behavior is motivated by more than one need level operating simultaneously. This theory included a frustration-regression process, in which inability to satisfy a higher need causes frustration and a regression to the level of need that is one step lower in a hierarchy of needs. ERG theory also suggested that the fulfillment of one need would enhance one’s desire. Process theories focused on the influence of subjective expectation, or the value that is placed on staff, on employees’ work efforts [27]. The crowding-out effect proposes that extrinsic rewards have a negative impact on intrinsic motivation [28]. Previous researches have suggested that both desired workplace incentives and perceived actual incentives affect job satisfaction, either directly or indirectly [23,29]. For example, Linz and Semykina assessed the relationship between job satisfaction and anticipated reward [29].

The purpose of this study was to assess the determinants of job satisfaction in community health workers in one Chinese province. A cross-sectional survey was conducted to measure actual and desired workplace characteristics and job satisfaction. Factor analysis was used to identify the main factors involved in workplace incentive. The key determinants of job satisfaction in community health workers were assessed, with particular attention directed toward actual and desired workplace characteristics.

## Methods

### Sample

A stratified cross-sectional survey of community health workers was conducted from October 1, 2012 to December 31, 2012. Three cities (Harbin, Suihua, and Qitaihe) were selected in order to account for the variability in regional per capita gross domestic product and healthcare development levels. Ten CHCs were randomly selected from each city. On average, 24 personnel, including administrative staff, general practitioners, public health physicians, nurses, rehabilitation doctors, dentists, and technicians worked in each of the selected CHCs. Seventy percent of employees were chosen randomly, excluding those who were absent. The research team visited each of the selected CHCs and invited all selected staff members to participate in the study. All of the subjects provided written informed consent to participate in the survey. Thereafter, a self-administered questionnaire was completed by all subjects. In total, there were 494 respondents; however, of these, 56 (11.3%) returned incomplete questionnaires. Therefore, the analysis file comprised 448 respondents.

### Measures

We refer to three main concepts in the study: first, workers’ desire for incentive is referred to as desired workplace characteristics (DWC); second, their perceptions of factors related to actual incentive were described as actual workplace characteristics (AWC); and third, we constructed a gap between desired and actual workplace characteristics and referred to it as GWC.

The survey questionnaire was composed of three sections. The first focused on the socioeconomic and demographic status of respondents.

The second was used to assess the value of actual and desired workplace characteristics using 44 potential workplace incentive items, such as opportunities to participate in decision making. These 44 items were chosen based on previous studies and a panel discussion that examined desired and perceived workplace incentives for health workers [10,17,18]. There were two aspects to each incentive item: desirability and perception. Respondents were required to assess their perceptions regarding AWC using a 5-point Likert-scale (1 = not good; 2 = slightly good; 3 = somewhat good; 4 = very good; and 5 = extremely good). Examples of the type of incentive item are listed in Table 1 [17,29]. The same items were used to assess the respondents’ DWC (see Table 1). The mean score for each subscale representing the GWC was calculated by subtracting the mean AWC score from the mean DWC score.

**Table 1:** **Questionnaire wording for actual and desired workplace characteristics**

		**Potential workplace incentive items for community health workers are listed below. Pleasecircle a number that best describes the extent to which each incentive item is reflected at your current workplace**					**Potential workplace incentive items for community health workers are listed below. Please circle a number that best describes the desirability of each incentive item**				
		1 = Not good to					1 = Not good to				
		5 = Extremely good					5 = Extremely good				
1	Opportunity for participating in decision-making	1	2	3	4	5	1	2	3	4	5
2	Appreciate policies for professional promotion	1	2	3	4	5	1	2	3	4	5
3	Fair competition mechanism	1	2	3	4	5	1	2	3	4	5
4	Good assessment and evaluation mechanism	1	2	3	4	5	1	2	3	4	5
		1	2	3	4	5	1	2	3	4	5

The third section of the questionnaire was used to assess job satisfaction, which can be assessed via a variety of methods including a number of questionnaires [30]. The 20-item Minnesota Satisfaction Questionnaire (MSQ) [31] was used to assess job satisfaction on a 5-point Likert-scale from 1 (very dissatisfied) to 5 (very satisfied). Overall job satisfaction was represented by a total of 20 items considered to be a composite of all of the facets of job satisfaction [31]. The scale achieved reasonable reliability in our sample (Cronbach’s α = 0.88) [32].

### Statistical analysis

There were four main components to the data analysis. First, descriptive statistics were reported for socioeconomic and demographic status, actual and desired workplace characteristics, and job satisfaction. Second, the underlying key dimensions of the DWC were assessed via a factor analysis of the 44 workplace incentive factors. The factor analysis was conducted via principal component analysis with varimax rotation [33]. In the first phase, nine incentive items with factor loadings of less than 0.4 or equally loaded factors on two subscales were eliminated. Subsequently, a second factor analysis was conducted, and eight moderately distinct and interpretable subscales were identified.

Third, multiple regression analysis was performed to identify the determinants of each subscale of DWC. Explanatory variables comprised AWC and the respondents’ characteristics.

Fourth, based on expectancy theory and previous research [17,29], four multiple regression models were used to identify the determinants of job satisfaction. Each regression model included the respondents’ characteristics. Model 1 assessed the impact of all subscales of AWC. Model 2 examined the role of the gap between desired and actual workplace characteristics (GWC). Model 3 assessed the independent roles played by both AWC and GWC in job satisfaction, while Model 4 examined potential interactions between AWC and GWC via inclusion of an interaction variable (AWC*GWC) for each subscale.

## Results

The socioeconomic and demographic status of respondents was examined. Subsequently, eight subscales of DWC were derived based on the factor analysis; for each of the eight subscales, DWC, AWC and GWC were measured, and the determinants of job satisfaction were assessed.

### Socioeconomic and demographic of respondents

Table 2 shows the socioeconomic and demographic status of respondents. The majority of respondents were female (75.4%), married (86.2%), and aged between 31 and 50 years (69.8%). More than 80% of respondents earned monthly incomes of less than 3,000 RMB (where $1.00 = 6.23 RMB in 2012). The majority of respondents (61.8%) were educated to a lower level than that of a college degree. Less than half of the respondents (42.4%) were middle professionals, and only 19.6% were senior professionals. Almost half of the respondents (47.3%) worked for a maximum of 40 hours per week.

**Table 2:** **Demographic characteristics for respondents**

**Demographic variables**		N*	**%**	**Demographic variables**		N*	**%**
Sex	Male	110	24.6	Age in years	21-30	92	20.6
	Female	338	75.4		31-40	152	33.9
Marital status	Married	386	86.2		41-50	161	35.9
	Others	62	13.8		>50	43	9.6
Occupation	Administrative staff	43	9.6	Years worked	≤5	84	18.8
	General practitioner	138	30.9		6-10	55	12.3
	Public health physician	105	23.4		11-15	57	12.7
	Nurse	122	27.2		16-20	66	14.7
	Other	40	8.9		≥21	186	41.5
Working hours (per week)	≤40	212	47.3	Monthly income (RMB)	<2000	186	41.5
	41-48	140	31.3		2000-2999	194	43.3
	49-56	62	13.8		3000- 3999	58	12.9
	>56	34	7.6		≥4000	10	2.3
Title	Senior	88	19.6	Educational background	High school or below	92	20.5
	Middle	190	42.4		Junior college	185	41.3
	Junior	144	32.1		College and above	171	38.2
	No title	26	5.9				

*N = 448.

### Identification of the main factors associated with desired workplace characteristics

Factor analysis yielded an eight-subscale structure that comprised a total of 35 items, as shown in Table 3. The eight-subscale solution accounted for 68.49% of the overall variance, and was found to be internally consistent (overall Cronbach’s α = 0.87). The subscales were renamed based on the conceptual meanings of the items [34] and comprised the following: system and policy; fringe benefits; work itself; working relationship; professional development; recognition; working environment; and remuneration, and accounted for 14.29%, 10.6%, 9.6%, 8.3%, 7.8%, 7.4%, 6.1%, and 4.6% of the overall variance, respectively. The Cronbach’s αs within individual subscales ranged from 0.80 to 0.91.

**Table 3:** **Factor analysis for desired workplace characteristics**

**Items of desired workplace characteristics**	**Subscales and loadings**							
	1*	2*	3*	4*	5*	6*	7*	8*
Appropriate policies for professional promotion	0.57							
Fair competition mechanism	0.65							
Opportunity for participating in decision-making	0.76							
Fair Assessment and Evaluation mechanism	0.80							
Simple rules and regulations	0.74							
Encouraging innovation	0.58							
Paid leave		0.70						
Home & health insurance		0.66						
Vacation days & public holiday gifts		0.78						
Special allowance		0.85						
Transportation subsidies or a commuter car		0.82						
Work being meaningful and important			0.61					
Clear division of tasks			0.63					
Workflow reasonable			0.71					
Independence from interference			0.75					
Congruence between work tasks and ability			0.60					
Support from coworkers				0.62				
Support from supervisors				0.73				
Good encouragement and communication				0.71				
Timely guidance need in work				0.59				
Congruence of requirement by different leadership				0.48				
Continuing educational opportunities					0.74			
On-the-job training					0.72			
Opportunity for position promotion					0.66			
Opportunity for career advancement					0.50			
Recognized and respected by community						0.58		
Recognized and appreciated by leaders						0.76		
Recognition for achievement						0.80		
Adequate equipment and infrastructure							0.68	
Adequate office resources							0.70	
Recreational facilities for workers							0.66	
Good maintenance of clinical equipment							0.57	
Stable income								0.61
Pay equity compared with others’ input/output ratio								0.75
Performance bonus								0.75
variance explained (%)	14.29	10.35	9.63	8.29	7.81	7.44	6.10	4.59
Eigenvalues	5.73	4.24	3.84	3.54	3.11	2.97	2.53	1.82
Cronbach Alpha	0.91	0.88	0.87	0.88	0.89	0.87	0.83	0.80

Notes: Extraction method: principal component analysis.

Rotation method: varimax with Kaiser normalization.

*1 = System & policy; 2 = Fringe benefits; 3 = Work itself; 4 = Working relationship;

5 = Professional development; 6 = recognition; 7 = Work environment; 8 = Remuneration.

### Analysis of desired and actual workplace characteristics and the gaps between these characteristics

Mean scores of subscales of DWC, AWC, and GWC are shown in Table 4. Desired remuneration (4.47), working environment (4.31), and professional development (4.30) ranked in the top three positions for DWC, while fringe benefits (3.99) were in the lowest position. Actual remuneration (2.03) was in the lowest position for AWC, while working relationships (2.90) ranked the highest position.

**Table 4:** **Descriptive analysis concerning facets of DWC**, **AWC and GWC**

**Workplace characteristics**	**DWC**			**AWC**			**GWC** **(= DWC** – **AWC)**			
	**Mean** *	**SD**	**Order**	**Mean**	**SD**	**Order**	**Mean** ^†^	**SD**	**Order**	**P**
Remuneration	4.47	0.62	1	2.03	1.07	8	2.44	1.26	1	<0.01
Work environment	4.31	0.60	2	2.72	0.81	3	1.58	0.92	4	<0.01
Professional development	4.30	0.64	3	2.63	0.64	5	1.67	0.81	3	<0.01
Working relationship	4.29	0.55	4	2.90	0.66	1	1.38	0.70	8	<0.01
System & policy	4.25	0.55	5	2.82	0.65	2	1.43	0.73	7	<0.01
Recognition	4.18	0.66	6	2.50	0.87	6	1.68	0.94	2	<0.01
Work itself	4.14	0.57	7	2.66	0.60	4	1.48	0.66	6	<0.01
Fringe benefits	3.99	0.74	8	2.49	0.82	7	1.50	1.09	5	<0.01

*Mean score of each subscale in DWC was calculated for each respondent by adding the value of each item belongs to the subscale of DWC and then divided by the numbers of the item.

^¶^Mean score of each subscale in AWC was calculated for each respondent by adding the values of each item belongs to the subscale of AWC and then divided by the numbers of the item.

^†^Mean score of each subscale of GWC was calculated by reducing mean score of AWC from mean score of DWC on each subscale.

A paired t test indicated that the mean score for each subscale of the AWC was significantly lower than the associated subscale of DWC (p <0.01). Remuneration was ranked in the highest position for GWC, followed by recognition, professional development, work environment, fringe benefits, work itself, system and policy, and work relationships.

### Regression for desired workplace characteristics

Eight multiple regression models were estimated and reported in Table 5 in order to identify the role of socioeconomic characteristics and AWC on each DWC subscale. The adjusted R^2^ ranged from 0.50 to 0.62. Results demonstrated that few socioeconomic characteristics were determinants of the DWC subscales.

**Table 5:** **Predictors of desired workplace characteristics**

**Desired workplace characteristics**								
**Variables**	**1 System & policy**	**2 Fringe benefits**	**3 Work itself**	**4 Working relationship**	**5 Professional development**	**6 Recognition**	**7 Working environment**	**8 Remuneration**
	**Beta**	**Beta**	**Beta**	**Beta**	**Beta**	**Beta**	**Beta**	**Beta**
**Occupation (Reference: administrative staff)**								
General practitioner	0.14**	0.17**	0.15**	0.12*	0.18*	0.15*	0.21**	0.08
Public health physician	0.26**	0.31**	0.21**	0.30**	0.21**	0.29*	0.35**	0.08
Nurse	0.03	-0.02	0.04	0.04	0.07	-0.01	0.11	-0.01
Other	0.15	0.02	0.11	0.03	0.14	0.04	0.28**	-0.05
**Sex (Reference: female)**								
Male	0.08	0.08	0.05	0.07	0.06	0.07	0.09	-0.02
**Marital status (Reference: single/divorced)**								
Married	-0.09	0.01	-0.09	-0.12	-0.11	-0.03	-0.09	-0.07
**Age in years (Reference: >50)**								
21-30	0.09	-0.10	0.25	0.23	0.17	0.15	0.41	0.11
31-40	0.28	0.17	0.41	0.39	0.32	0.38	0.51	0.44
41-50	0.32	0.06	0.34	0.38	0.17	0.39	0.45	0.40
**Educational Background (Reference: High school or below)**								
Junior college	-0.03	0.04	0.02	0.03	0.05	0.01	-0.02	0.120
College and above	-0.09	0.02	0.01	-0.00	0.08	-0.01	-0.05	0.104
**Years worked (Reference: ≤5)**								
6-10	-0.07	-0.21	-0.25**	-0.18	0.29**	-0.23	-0.14	-0.07
11-15	-0.00	-0.18	-0.18*	-0.14	0.23*	-0.21	-0.05	-0.01
**Desired workplace characteristics**								
16-20	-0.07	-0.25	-0.13	-0.12	0.16	-0.16	-0.17	-0.13
>20	0.01	-0.06	-0.07	0.02	0.03	-0.06	-0.05	-0.04
**Weekly hours worked (Reference: ≤40)**								
41-48	-0.04*	-0.02	-0.00	-0.02	-0.06	-0.03	-0.06	0.04
49-56	-0.06*	-0.03	-0.00	-0.03	-0.09	0.00	-0.10	0.07
>56	-0.01*	-0.06	-0.04	-0.00	-0.04	0.05	-0.03	0.16
**Monthly income in RMB (Reference: <2000)**								
2000-2999	-0.06	-0.13*	-0.06	-0.09*	-0.02	0.09	-0.08	-0.14*
3000- 3999	-0.05	-0.10	-0.07	-0.07	-0.04	0.14*	-0.09	-0.20*
>4000	-0.18	-0.11	-0.20	-0.27*	-0.05	0.37*	0.02	-0.54**
**Actual workplace characteristics**								
System & policy	-0.61**	-0.06	-0.05	-0.02	-0.13**	0.08	-0.011	-0.08
Fringe benefits	-0.13**	-0.54**	-0.15	-0.14**	-0.13**	-0.17**	-0.10	0.01
work itself	0.09*	0.03	-0.38**	-0.16**	0.00	0.03	0.00	-0.01
working relationship	-0.04	-0.03	0.01	-0.67**	0.06	0.02	0.02	-0.03
Professional development	0.06	0.02	-0.08**	-0.08**	-0.38**	0.07*	0.11**	0.03
Recognition	0.05**	-0.01	0.00	-0.06**	0.05	-0.65**	0.08**	0.02
Working environment	-0.00	-0.03	-0.09**	-0.04	-0.02	0.00	-0.70**	0.02
Remuneration	-0.00	0.43**	0.00	0.00	-0.015	-0.00	0.01	-0.70**
Adjusted R2	0.52	0.57	0.46	0.53	0.47	0.62	0.62	0.59

*p < 0.05, **p < 0.01.

Consistent with ERG theory [26] and the crowding-out effect [28], we found that some subscales of AWC influenced DWC subscales either positively or negatively System and policy and fringe benefits in AWC were significant positive predictors of desired system and policy, while work itself and recognition in AWC were significant negative predictors in regression 1. Actual fringe benefits were negative predictors and actual remuneration was a positive predictor of desired fringe benefits in regression 2. Regression 3. showed that actual fringe benefits, work itself, and working environment were negative predictors of desired work itself. In regression 4. actual fringe benefits, work itself, working relationships, work environment, and recognition were negative predictors of desired working relationship. Actual system and policy, fringe benefits, and professional development were negative predictors of desired professional development in regression 5. Regression 6. indicated that fringe benefits and recognition in AWC were negative predictors of desired recognition. In regression 7. actual fringe benefits and working environment were negative predictors, while actual professional development and recognition were positive predictors of desired working environment. In regression 8, actual remuneration was a negative predictor, while actual fringe benefits were positive predictors.

### Regression for overall job satisfaction

The mean value of intrinsic satisfaction was 3.79, which was higher than those for external (3.50) and overall satisfaction (3.69). Table 6 shows four models used to assess the key determinants of overall job satisfaction. Occupation and years worked were both determinants in these four models. In Model 1, inclusion of the subscales of AWC resulted in occupation, actual system and policy, work itself, and working relationships as positive predictors of overall job satisfaction (adjusted R^2^ = 0.37). In Model 2, inclusion of the subscales of GWC resulted in occupation, age, system and policy, fringe benefits, working relationship, professional development, and remuneration of GWC as significant negative predictors of overall job satisfaction (adjusted R^2^ = 0.62). In Model 3, inclusion of the subscales from both AWC and GWC resulted in an adjusted R^2^ of 0.62. None of the AWC subscales were significant, and F-joint test results did not promote rejection of the hypothesis stating that this group of variables displayed coefficients of zero (F = 1.16, P = 0.32). Model 4 augmented Model 3 through inclusion of the interaction term, AWC*GWC. None of the subscales of the interaction term were significant, and F-joint test results did not promote rejection of the hypothesis stating that this group of variables displayed coefficients of zero (F = 0.73, P = 0.66).

**Table 6:** **Predictors of overall job satisfaction**

**Variables**	**Model 1**	**Model 2**	**Model 3**	**Model 4**
	**Beta**	**Beta**	**Beta**	**Beta**
**Occupation** **(Reference:** ** Administrative staff)**				
General practitioner	-0.00	0.13^*^	0.12	0.12
Nurse	-0.19	0.07	0.05	0.05
Public health physician	0.22^*^	0.24^*^	0.24^**^	0.23^**^
Other	0.02	0.09	0.06	0.08
**Sex** **(Reference:** **female)**				
Male	-0.08	-0.02	-0.03	-0.04
**Marital status ** **(Reference:** **Single/** **divorced)**				
Married	-0.05	-0.11	-0.12	-0.10
**Age in years ** **(Reference: >** **50)**				
21-30	-0.07	0.00	0.04	0.02
31-40	-0.14	0.14	0.18	0.16
41-50	-0.20	0.03	0.07	0.05
**Educational background** **(Reference:** **High school or below)**				
Junior college	-0.03	-0.01	0.00	-0.00
College and above	-0.01	-0.02	0.00	-0.00
**Years worked ** **(Reference: ≤** **5)**				
6-10	-0.12	-0.15	-0.15	-0.15
11-15	-0.15	-0.36^**^	-0.35^**^	-0.31^*^
16-20	0.00	-0.16	-0.15	-0.12
>20	-0.11	-0.27	-0.27^**^	-0.26^*^
**Weekly hours worked ** **(Reference: ≤** **40)**				
41-48	0.00	-0.03	-0.03	-0.02
49-56	0.04	-0.00	-0.01	0.01
>56	0.06	0.03	0.02	0.02
**Monthly income in RMB** **(Reference: <** **2000)**				
2000-2999	0.13	0.02	0.03	0.04
3000- 3999	0.13	0.01	0.02	0.05
>4000	0.17	0.06	0.08	-0.04
Variables	Model 1	Model 2	Model 3	Model 4
	Beta	Beta	Beta	Beta
**Subscales of AWC**				
System & policy	0.18^**^		0.13	0.35
Fringe benefits	0.26^**^		0.06	0.02
Work itself	0.00		0.04	0.21
Working relationship	0.21^**^		0.01	-0.14
Professional development	0.00		-0.02	0.09
Recognition	-0.02		-0.07	-0.15
Working environment	-0.02		-0.02	-0.08
Remuneration	0.02		-0.08	-0.10
**Subscales of GWC**				
System & policy		-0.12^*^	-0.00	0.09
Fringe benefits		-.26^**^	-0.20^**^	-0.21^*^
Work itself		-0.09	-0.04	0.03
Working relationship		-0.30^**^	-0.29^**^	-0.36^**^
Professional development		-0.18^**^	-0.19^*^	-0.12
Recognition		-0.04	-0.11	-0.16^*^
Working environment		0.06	0.03	-0.00
Remuneration		-0.09^**^	-0.16^**^	-0.18^*^
**Subscales of AWC** ^*^ **GWC**				
System & policy				0.06
Fringe benefits				-0.01
Work itself				0.05
Working relationship				-0.04
Professional development				0.03
Recognition				-0.02
Working environment				-0.01
Remuneration				-0.00
Adjusted R^2^	0.37	0.62	0.62	0.63

^*^p < 0.05, ^**^p < 0.01.

## Discussion

This study was one of the most recent efforts to focus on gaps between workers’ desires and perceptions of workplace incentives and analyze the determinants of job satisfaction in urban community health workers in China. An eight-subscale structure of workplace characteristics was derived via factor analysis. The mean value of each DWC subscale was higher than the associated AWC subscales, and some AWC subscales predicted DWC, either positively or negatively. We also found that five GWC subscales and some socioeconomic characteristics significantly predicted job satisfaction.

In this study, overall job satisfaction was higher than extrinsic job satisfaction and lower than intrinsic job satisfaction. This finding is consistent with previous research on job satisfaction in Chinese community health workers [35]. To account for variations in job satisfaction, four distinct multiple regression models were assessed, with specific consideration of AWC and GWC. The socioeconomic characteristics of workers were included in all models, and each model accounted for a significant proportion of the variation in job satisfaction. While the AWC subscales were significant when used alone (Model 1), which replicated previous research [17,29], this model was dominated by Model 2, which only used GWC subscales. In this context, occupation, years worked, and five subscales (system & policy; fringe benefits; working relationship; professional development; and remuneration.) were significant determinants. The result was quite similar to those of previous studies [12,14]. When AWC and GWC were combined (Model 3), none of the individual AWC subscales were significant determinants of job satisfaction. Further, the interaction between AWC and GWC was included in Model 4, and while the subscales of both the interaction variable (AWC*GWC) and the AWC accounted for a larger proportion of the variance, none were significant individually. Therefore, Model 2 was the superior model. This was consistent with Longest’s study, which indicated that only needs that were not yet fulfilled influenced behavior [36].

These findings have significant implications for policy makers and CHC managers in their efforts to improve workers’ job satisfaction. First, they should pay more attention to narrowing the gaps between the AWC and DWC, as five subscales of the GWC were negative predictors of job satisfaction. These findings were consistent with Locke’s study, which linked job satisfaction with discrepancy between actual and expected rewards and facets of the job [37]. The results also indicated that there was considerable room for improvement in narrowing these gaps in order to improve job satisfaction, as the mean scores for each subscale of the AWC were significantly lower than those of the associated DWC subscale. Longest’s study proposed that individuals are beings of desire, whose needs depend on what they already have [36]. ERG theory suggested that the fulfillment of one need would enhance one’s desire for a higher-level need [26].

In the present study, remuneration (stable income, pay equity, and performance bonus) ranked highest for DWC and GWC [29]. According to Herzberg’s motivation-hygiene theory, remuneration is a hygiene factor that is required to ensure that an employee is satisfied. Previous studies have revealed that adequacy of pay and perceived equity via others influenced job satisfaction and behavior [38]. Studies in most developing countries have shown that job dissatisfaction in health workers is primarily accounted for by low salaries [16,39]. Therefore, managers should provide fair pay to reduce the gap between desired and actual remuneration to improve job satisfaction [40,41].

Second, financial incentives are not the sole means of stimulating motivation and improving job satisfaction [42,43]. Other factors, particularly working relationships, professional development, and system and policy subscales of GWC, were negative predictors of job satisfaction [44,45]. These findings were congruous with previous studies and highlighted the importance of packaging financial and nonfinancial incentives [46,47]. In addition, the regression equation for DWC showed that general practitioners, public health physicians, and workers with 6–15 years in the profession expressed greater desire for professional development.

Third, managers and policymakers should consider DWC and AWC comprehensively, as subscales of AWC can influence subscales of DWC positively or negatively. In our study, we found that actual remuneration was a positive predictor of desired fringe benefits, and actual fringe benefits were a positive predictor of desired remuneration. Consequently, an increase in actual fringe benefits would increase the gap between desired remuneration and actual remuneration. Similarly, an increase in actual remuneration would enlarge the gap between desired and actual fringe benefits. Therefore, to improve job satisfaction, care should be taken to balance the relationship between remuneration and fringe benefits.

Results also revealed that the working relationship subscale received the lowest ranking in GWC and the highest ranking in AWC. Three reasons accounted for this result. First, CHC is a simply structured organization employing fewer workers relative to general hospitals; therefore, managers enjoy greater opportunity to tailor incentives to individual staff members and provide employees with timely guidance if required. Second, it was easier for employees to communicate and form positive relationships in CHCs [16,48,49]. Third, as ERG theory indicates, if a higher-level need appears too difficult to fulfill, the person may regress to lower-level needs [26]. In our study, actual remuneration and fringe benefits were both low-level needs; therefore, financial needs increased, and desire for working relationships decreased. Therefore, working relationships were ranked in the lowest position in GWC.

In this study, we found that general practitioners and public health physicians reported higher job satisfaction compared with administrative staff. In community health institutions, general practitioners and public health physicians were the main providers of basic public health and medical services. They earn higher salaries and would be afforded more opportunities to join conferences and receive in-job training. The results also indicated that health workers who had worked in the position for 11–15 years displayed lower levels of job satisfaction. Most of these workers faced difficulties with respect to promotion, because it was difficult for community health workers to achieve promotion, as there were limited annual promotion quotas in CHCs in Heilongjiang Province. This would exert a negative influence on job satisfaction.

### Limitations

The findings in this study should be viewed in light of two key limitations. First, the instrument used to assess actual and desired workplace characteristics was not an established scale, and its content validity and reliability has yet to be examined comprehensively. In this study, the process of constructing the instrument was guided by multiple standards of actual and desired workplace characteristics available worldwide and items developed in earlier studies [10,17,18]. Several panel reviews involving associated researchers, health care managers, and community health workers were conducted in order to establish content validity. In addition, factor analysis and principal component analysis were used to develop an internally consistent scale and reduce items [17,50]. Based on these methods, the study achieved good internal consistency (Cronbach’s α = 0.87). Moreover, the eight-subscale solution accounted for 68.49% of the overall variance, which indicated that the measurement instrument displayed reasonable validity.

Second, this study was based on a small sample of community health workers, which may limit the generalizability of the research findings. A multistage, stratified sampling design was used to ensure that study data were provincially representative. Three sample cities were selected to account for the variability in regional per capita gross domestic product, and the level of healthcare development and 10 CHCs in each city were selected randomly. The proportions of administrative staff, general practitioners, public health physicians, nurses, and others in this study were close to proportions in the wider provincial population [1]. Consequently, this sample was representative of Heilongjiang community health service providers, which enhanced the generalizability of the study findings.

## Conclusions

It is important that health-care managers and policy decision makers improve and maintain the job satisfaction of health workers in low-resource settings. We analyzed the relationship between desired and actual workplace characteristics comprehensively, and the key determinants of job satisfaction were assessed using multiple regression analysis. The results indicated that subscales of AWC affect subscales of DWC both positively and negatively. Five subscales of GWC, comprising system and policy, fringe benefits, working relationship, professional development, and remuneration were significant negative predictors of job satisfaction. The study findings suggest that managers should endeavor to reduce the gap between DWC and AWC to improve job satisfaction. The results also suggest that some subscales representing the gaps (i.e., between DWC and AWC) are more important than others as determinants of job satisfaction. Two methods could be used to reduce this gap. One is to improve actual workplace incentives, and the other is to dampen DWC. It is difficult for managers to help every staff member to meet all of their needs, due to limited resources. However, subscales of AWC predicted subscales of DWC both positively and negatively. Therefore, in order to formulate incentives suitable for their own organizations, managers should consider determining which incentives they can provide and which workers desire, in addition to examining the relationship between workplace characteristics and job satisfaction.

### Ethical considerations

Human subjects’ approval was gained from the Medical Ethic Committee of Harbin Medical University. The data were collected anonymously. Respondents were assured that participation in this survey was voluntary, with the return of completed questionnaires being taken as consent to participate.
